# Preparation of Chloral

**Published:** 1871-05

**Authors:** E. R. Squibb


### ARTICLE VII.

### *On the Preparation of Chloral.*

Extracted from the remarks of Dr. E. R. Squibb, before the American Pharmaceutical Association.

Chloral is the ultimate product of the action of chlorine on alcohol, as its name implies, the first syllables of the two words being formed into the name: "chlor," the syllable of chlorine, and "al," the first syllable of alcohol, making "chloral." When chlorine gas in a dry state is passed into absolute alcohol, a series of changes appear to take place, which may depend on the abstraction of hydrogen and the substitution of chlorine. The first portions of chlorine gas that pass into absolute alcohol are converted, or appear to be converted, at once into hydrochloric acid, and that hydrochloric acid is absorbed by the remainder of the alcohol, and reacts with it, producing hydrochloric ether. The second step in the reaction is to again decompose or supersaturate this hydrochloric ether with chlorine, and then hydrochloric acid escapes and a substitution appears to take place, whereby chlorine is substituted for hydrogen in the already decomposed alcohol. This is but a rude outline of the process. Chloral was discovered by Liebig in 1829 or 1830, although the paper in which it was described was not published until about 1832, therefore it is commonly stated that he discovered it in 1832, which is incorrect. Dumas was the next who investigated it, and these two observers investigated it as a table specimen product. Last year, Dr. Otto Liebreich, in his physiological investigations regarding the group of anæsthetic chemicals, reasoned back to this substance the known effects of chloroform, and tried it first upon animals, then upon patients. At first he supposed it was an anæsthetic, but afterwards modified this view, and now I believe regards it as a hypnotic, and, in some cases, an anodyne. The apparatus for making chloral consists, first, in the means of generating chlorine ; second, in the means of drying the chlorine ; third, in the means of passing it into absolute alcohol without loss ; and, fourth, having the absolute alcohol in such a position that it can be gradually warmed. The process requires about twenty-eight days for the current of chlorine to be passed into the absolute alcohol, and I believe the slower the current passes into the absolute alcohol the better ; that is to say, the longer the time which is taken to produce the chloral the better ; I think there is less waste and more chloral obtained for the same quantity of alcohol. It is a curious circumstance that hydrate of chloral is produced by passing the chlorine into absolute alcohol, and this shows that water is one of the results of the decomposition of the alcohol ; yet if hydrated alcohol be used, the product is different. I have tried different degrees of strength of alcohol, from absolute down to ninety-two per cent., and have obtained good results only from absolute alcohol. 16 gallons of such alcohol, in twenty-eight days, with the use of about a ton and a quarter of mixture of binoxide of manganese and common salt, and about the same quantity of sulphuric acid,—the 16 gallons of absolute alcohol weighing abut 92 pounds—I obtained about 160 pounds of crude hydrate of chloral. This crude hydrate of chloral, as it is made by the passage of the chlorine into the alcohol, is contaminated with several other products which pass over in the distillation, and cannot be separated by simple distillation. It is necessary, therefore to apply sulphuric acid in the purification of the chloral. Concentrated sulphuric acid is shaken with the crude hydrate of chloral, and the dehydrated chloral is then distilled off from the sulphuric acid. In this way we get chloral that is free from water. After purifying this by one or two applications of sulphuric acid, then the stœchiometric proportion of water is added, and it is either sublimed or crystallized. In connection with this hydrature allow me to go back to the name of chloral. I propose to call it simply chloral,—not hydrate of chloral, nor chloral hydrate. It seems to me surplusage, as we do not in our language commonly call hydrated compounds hydrates; that is, we do not usually recognize the presence of combined water in the names of chemical compounds.

We do not say hydrate of sulphuric acid, or hydrate of hydrochloric acid, and in this case we shall save a good deal of nomenclature that is useless by calling it simply chloral. We heard yesterday, that the bees by taking a little honey from each flower gathered thirty millions of pounds. Every flower and every bee helps to make the aggregate. A certain amount of nervous force is expended on every word we utter, and we save this word now (and now is the time to start), it will save an aggregate of nervous force which, in the future, will amount to a great many lives. I don't believe in useless langauge, particularly where it can so well be avoided, and therefore, think we had better call this from the beginning, simply chloral, although the other name is pretty generally used.*

The difficulties in the way of making chloral are very numerous. The apparatus I have now at work is about the tenth modification from the first one, and I started with all the knowledge on the subject then in the books. The liberation of chlorine from common salt and black oxide of manganese by running acid sulphuric into it is easy enough, but unless the current be steady the result is imperfect, and there can be no good or definite calculations made as to the time or the quantity. The black oxide of manganese and common salt need both to be assayed and added together in their equivalent proportion, and then the calculated amount of sulphuric acid in any given specimen is to be made upon its specific gravity, and acid can only be added to the mixture by calculation, because, if added until chlorine ceases to be eliminated a great excess will be used. I mix 100 pounds of the mixture of black oxide of manganese and common salt with about ten gallons of water in a still, and then run seven gallons of 60° slowly into it, using "pan acid," 1,562 specific gravity, using a mechanical stirrer, and heating the mixture. In this a tolerably uniform current of chlorine is eliminated. This is then conducted to the drying apparatus, which consists of a three-neck Woulfe's bottle, with a long, narrow glass percolator ground into the middle neck. This percolator is filled with pieces of broken glass from which the fine particles have been sifted out, and into the top of this broken glass, concentrated sulphuric acid is supplied from an elevated reservoir. This acid percolates through the broken glass, and accumulates in the Woulfe's bottle below until it reaches the level of an adjusted syphon, by which it is dicharged through one of the necks of the

---

*While this note is being prepared for publication a serious mistake by abbreviating the words Hydrate of Chloral "Hyd. Chlor." in prescription, was corrected in time to avoid danger.—E. R S.

bottle. Thus the gas is made first to bubble through the acid in the bottle, and then to pass over the extended surface of broken glass in the tall percolator, this surface being kept moistened with fresh portions of acid, and thus becomes thoroughly dried and in the proper condition to enter the alcohol. The chlorine thus passed down into the alcohol at first increases the volume of alcohol by one-fourth. At first, the whole of the bubbles of gas are absorbed, and the alcohol increases in volume and becomes heated, the bottle requiring to be kept cold; but after about three days the reaction between the chlorine and alcohol becomes more sluggish, and then a little heat in the bath is necessary. From that time the bath is made gradually warmer until the end of the process, which is determined by the gas pressing unchanged through hot liquid in the bottles. The product is then the crude hydrate of chloral. Then if the contents of the bottles be allowed to cool a large proportion crystallizes. It will run from one part of the bottle to another, but still is very moist. This is taken in portions of about twenty pounds at a time and shaken up with six to eight pounds of sulphuric acid, the whole mixture poured into a tubulated retort and the chloral distilled off. This is received in a clean, dry vessel, is weighed, and then partially hydrated with a weighed quantity of water. Carbonate of lime and slacked lime are then added in the proportion of four ounces to each twenty pounds, and the mixture is again distilled from a clean apparatus. The result of the distillation now is partially hydrated chloral; it distils better partially hydrated than when hydrated entirely. The remainder of the water required by stœchiometrical calculation is now added, and the hot liquid poured on plates to crystallize, the plates being covered by bell glass. In a few hours the crystallization is complete, and if well managed the contents of the plates is in a solid cake, which is rubbed into a coarse, damp powder in a clean mortar, and filled into bottles.

I obtained from 16 gallons of absolute alcohol, 160 pounds of crude chloral, which, when purified, yielded in the neighborhood of 125 pounds of purified hydrate of chloral. That is about the best yield I have had. I have now about 65 gallons in process all the time, by a series of baths, by which I expect to get 140 pounds, or thereabouts, every week, or every ten days'; that is, each bath being of a different age, and being finished in about thirty days, will give one bath or process every ten days. It will thus be seen that it is not a very profitable preparation to make, particularly when made in competition with the German article, and I believe I should never have undertaken to make it, except for my conviction that it is the most important of all the additions to the materia medica for many years past, and very commonly sent to our market from abroad of very bad quality, and without any traceable responsibility in regard to quality or make.

Some accidents of an apparently trivial nature seemed to indicate that chloral is very liable to decomposition from contact with organic matter, but experiments have shown that it is not equally liable to this decomposition from all kinds of organic matter. Even the same kind of organic matter does not always produce the same effect with the same chloral. For example, where syrup of orange-peel is used as a vehicle, decomposition, with the production of hydrochloric acid, will sometimes commence in a day or two, and sometimes not for weeks, though the apparent conditions be the same. One observer will testify that with simple syrup it never spoils or decomposes, while another equally trustworthy, will find the same chloral decompose with simple syrup very promptly. Under such circumstances, the only safe practice is to keep chloral as free as possible from all organic matter until we know more about it; and this particularly in view of the harm it does when given in even a partially decomposed solution. It appears to be by far the best practice to dispense it in simple watery solution in glass-stopped vials, since in this condition it keeps indefinitely, and can be added to any desired vehicle at the time of taking. And ice-water appears to be about as good a vehicle for this, as for all saline substances, as any yet devised. When given to patients who have been long fasting it is often found to disagree with them, or at best to affect them less favorably than when given near a meal, or when the gastric secretions are not in the condition of long fasting. Hence, the syrup of orange-peel, or the mucilage, &c., with which it is common to give it, may not be without useful effect, and those physicians who have now abandoned these mixtures for the simple solution, often if not generally, advise their patients to eat a cracker, or take some other light food in small quantity, before or immediately after an hypnotic dose. When the medicine affects persons unfavorably, it should always be examined for hydrochloric acid by smelling and tasting, and by litmus paper. Nitrate of silver is too sensitive a test, for if the solution has been some time made, and especially when water containing organic matter is used, a cloudiness may be produced with this test which it is quite safe to disregard.—*The Pharmacist.*

